# Wavelength-Tunable Single-Mode Microlasers Based on Photoresponsive Pitch Modulation of Liquid Crystals for Information Encryption

**DOI:** 10.34133/2020/6539431

**Published:** 2020-12-02

**Authors:** Fa-Feng Xu, Zhong-Liang Gong, Yu-Wu Zhong, Jiannian Yao, Yong Sheng Zhao

**Affiliations:** ^1^Key Laboratory of Photochemistry, Institute of Chemistry, Chinese Academy of Sciences, Beijing 100190, China; ^2^University of Chinese Academy of Sciences, Beijing 100049, China

## Abstract

Information encryption and decryption have attracted particular attention; however, the applications are frequently restricted by limited coding capacity due to the indistinguishable broad photoluminescence band of conventional stimuli-responsive fluorescent materials. Here, we present a concept of confidential information encryption with photoresponsive liquid crystal (LC) lasing materials, which were used to fabricate ordered microlaser arrays through a microtemplate-assisted inkjet printing method. LC microlasers exhibit narrow-bandwidth single-mode emissions, and the wavelength of LC microlasers was reversibly modulated based on the optical isomerization of the chiral dopant in LCs. On this basis, we demonstrate phototunable information authentication on LC microlaser arrays using the wavelength of LC microlasers as primary codes. These results provide enlightenment for the implementation of microlaser-based cryptographic primitives for information encryption and anticounterfeiting applications.

## 1. Introduction

Information encryption and decryption have attracted tremendous interest for tightly protecting significant information, offering many advanced applications ranging from high-throughput data storage to secure communication [1, 2]. The ever-increasing demands for high-level information security call for appropriate primitive systems capable of not only convenient information encryption but also efficient data authentication. Stimuli-responsive fluorescent materials have found their successful applications as data recording and document encryption systems owing to their tactfully changed luminescent outputs in response to external stimuli [3–5], which prevent the secret information from being stolen, mimicked, or forged. To date, various smart luminescent materials, including semiconductor nanocrystals [6, 7], dyes [8], lanthanide-doped nanoparticles [9, 10], carbon dots [11, 12], and transition-metal complexes [13, 14], have been explored. However, spontaneous emission from such materials usually produces broad photoluminescence (PL) bands, which are prone to be overlapped and result in the limited coding capacity [15–17]. Stimulated emission, which can generate discrete narrow lasing signals for easily distinguishable readout [18, 19], shows greater potential in the security field than conventional spontaneous emission [20–22]. Therefore, exploiting appropriate lasing systems as cryptographic primitives is of great significance for the implementation of high-security information encryption and decryption.

Liquid crystal (LC) materials with natural photonic crystal superstructures and excellent doping flexibility are promising candidate for the development of narrow-bandwidth lasers [23, 24], which allow for potential encryption applications as cryptographic primitives. Moreover, as a flexible organic lasing system [25–27], LCs possess outstanding processability and can be fabricated into ordered microlaser arrays [28–30], affording an opportunity for high-throughput parallel encryption. Further, the helical pitch of LCs and corresponding wavelength of LC lasers can sensitively respond to external stimuli, such as thermal stimuli [31, 32], electrical stimuli [33, 34], and mechanical stimuli [35], which facilitates the fulfillment of stimuli-responsive confidential encryption. Thus, external field-responsive LC lasing systems would be an ideal platform for confidential information protection.

In this work, we present a concept of confidential information encryption using photoresponsive LC lasing materials, which were used to fabricate ordered microlaser arrays through a microtemplate-assisted inkjet printing method. Single-mode LC microlasers were obtained with LC superstructures serving as distributed feedback cavities and embedded luminescent dye providing optical gain. Optically controlled isomerization of chiral dopants regulated the helical pitch of LCs and permitted reversible modulation of the wavelength of LC microlasers. On this basis, we demonstrate phototunable information encryption and authentication on LC microlaser arrays using the wavelength of LC microlasers as primary code. These results open a new avenue for microlaser-based information encryption and will stimulate fascinating cryptographic applications.

## 2. Results

**Figure 1:**
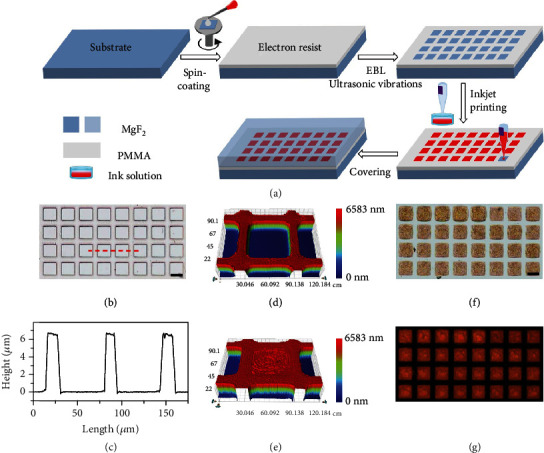
Fabrication procedures of a LC microlaser array. (a) Schematic diagram for the fabrication process of the LC microlaser array. (b) Optical microscopy image of polymeric microtemplates. (c) Plot of the height *vs.* the length of the microtemplates (marked as the dashed red line in (b)) by a step profiler. Scale bar: 50 *μ*m. Three-dimensional profiles of a microtemplate filled (d) without and (e) with LC-contained ink solutions by a contourgraph. (f) Bright-field and (g) dark-field microcopy images of the LC microlaser array. Scale bar: 50 *μ*m.

LC microlaser arrays were prepared by precisely depositing LC-contained ink solutions into polymeric microtemplates in regular alignment on substrates according to the predesigned digital patterns. As schematically illustrated in Figure 1(a), the fabrication procedure includes the preparation process of the polymeric microtemplates by etching the spin-coated film of the electron resist (polymethyl methacrylate (PMMA)) on the MgF_2_ substrate via electron beam lithography (EBL) that is a robust and effective technique for the production of microdevices, the printing process of depositing LC-contained ink solutions into the as-prepared microtemplates assisted by ultrasonic vibration, which is widely applied in the field of microelectronics, and the encapsulation process of covering another MgF_2_ wafer onto the ink-filled microtemplates. The as-fabricated polymeric microtemplates with microscale dimensions and depths (Figure 1(b)–(d)) can tightly confine the deposited ink solutions. The deposited ink solutions perfectly filled the microtemplates to produce individual microunits (Figure 1(e)), which can be precisely controlled by altering the vibration strength in the printing process (Fig. S1; see Materials and Methods). The structural parameter of the microunits, including shape (Fig. S2) and size (Fig. S3), can be rationally modulated by varying the microtemplates with the same parameters. The fabricated microunits were regularly patterned into an ordered emissive array (Figure 1(f) and (g)) according to the well-organized geometry of the microtemplates, which serve as potential microlaser-based cryptographic primitives for information encryption applications.

**Figure 2:**
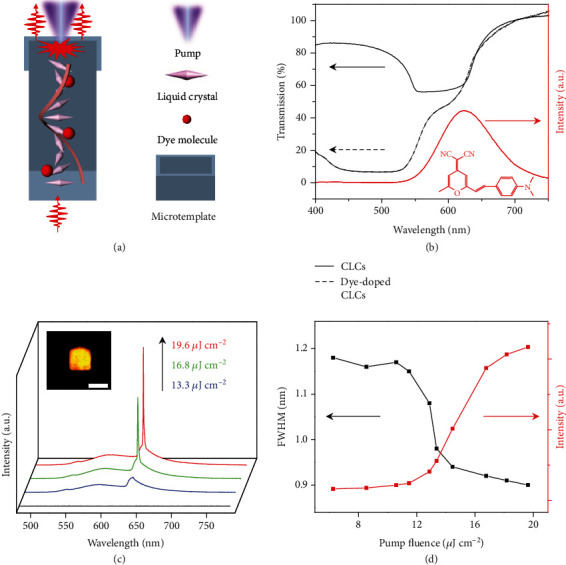
LC lasing from the printed microunit. (a) Sketch of an individual LC microlaser. (b) Transmission spectra of the CLCs doped with and without the dye and PL spectrum of the dye in ethanol. The concentration of the dye is 0.01 mM. Inset: the molecular structure of the dye. (c) PL spectra of an individual microunit under different pump fluences. Inset: PL image of the corresponding microunit. Scale bar: 50 *μ*m. (d) Plots of the PL peak intensity and the FWHM of the microunit *vs.* the pump fluence.

Upon pulsed illumination, effective lasing from the as-fabricated microunits would be expected with LC superstructures serving as photonic crystal cavities and embedded dyes providing optical gains, as depicted in Figure 2(a). In the microunits, through introducing chiral dopants into the LC matrix, cholesteric LC (CLC) superstructures were obtained since their pitches were captured and recorded in SEM images (Fig. S4) by using the UV curing method [36], which can serve as photonic crystal cavities to support laser oscillations at the band edges [37]. It should be noted that there is no alignment treatment of the substrate adopted here as it has been demonstrated not necessarily needed [28] and the formation of helical structures of CLC in the microunits probably can be ascribed to the induction of polymeric microtemplates [30]. The luminescent dye, 4-(dicyanomethylene)-2-methyl-6-(4-dimethylaminostyryl)-4H-pyran (Fig. S5), with PL emissions matching the long-wavelength band edge of the CLCs (Figure 2(b)), was doped into the LC solutions, which did not lead to obvious shift of the band edge and thus ensures lasing at the designed wavelength.

When the as-fabricated microunit was uniformly excited by a pulsed laser beam (490 nm, ~150 fs, and repetition rate of 1 kHz) in a custom microphotoluminescence system (Fig. S6), evidently modulated PL spectra of the microunit were observed, with much higher PL intensities at the band edges than that inside the bandgap (Fig. S7). Such unique modulation of PL emissions can be attributed to the fluctuation of density of optical states (DOS) at different positions of photonic bands of the CLCs [38], and the resulted higher DOS at band edges can favor the generation of low-threshold laser emissions [39]. With increasing pump fluence, the PL intensities of the peak at ~637 nm, where the long-wavelength band edge of the CLCs locates, were dramatically amplified (Figure 2(c)). The plot of the corresponding PL peak intensity as a function of the pump fluence exhibited a clear knee behavior characteristic at the threshold of ~13.2 *μ*J cm^−2^ (Figure 2(d)), which confirmed the lasing action of the microunit. Above the onset power, the full-width at half-maximum (FWHM) of the PL peak sharply narrowed down to ~0.9 nm (19.6 *μ*J cm^−2^), which revealed a microcavity effect with a quality factor of ~703 (defined as *λ*/FWHM). Such narrow PL peaks are in stark contrast with broadband PL peaks of the dye in tradition matrix, which undergo spontaneous emission mechanisms (Fig. S8). Note that the experimental result of narrow peaks based on obvious broadband fluorescence backgrounds reflects that the stimulated emission merely dominates due to the good arrangement of LCs induced by microtemplates, which can be further improved via alignment treatment thus excellent lasing emission with flat backgrounds would be expected [23, 29]. Single-mode lasing from the microunit was obtained due to the spatially modulated refractive index from CLC superstructures, which can be easily distinguished without confusion [40–42], and thus benefits the acquisition of lasing wavelength-based information encryption.

**Figure 3:**
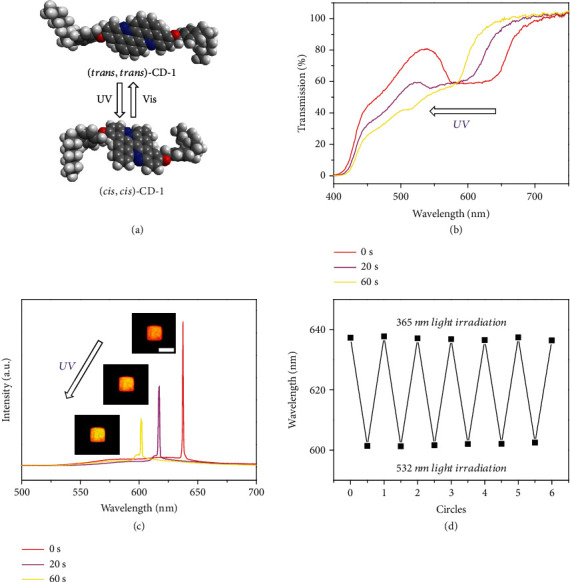
Phototunable lasing of the microunit. (a) Schematic illustration of the phototunable *trans*-*cis* isomerization process of CD-1. (b) Transmission spectra of CD-doped LCs under UV (365 nm, 20 mW cm^−2^) illumination with 0 s, 20 s, and 60 s. (c) PL spectra of the microunit under temporal UV illumination. Insets: PL images of the corresponding microunit. Scale bar: 50 *μ*m. (d) Plot of the lasing wavelength (637 nm and 601 nm) against the switching cycles. In each cycle, the microunit is illuminated by a UV light (365 nm, 20 mW cm^−2^) for 60 s and a visible light (532 nm, 20 mW cm^−2^) for 2 m.

The lasing wavelength of the as-fabricated microunits can be effectively modulated through varying the lattice constant of the one-dimensional photonic crystal superstructures of the CLCs (the helical pitch), as the pitch determines the position of the photonic band and thus affects the resonant band edge mode [43]. The photoisomerization of chiral dopants (CDs) in LC matrixes has been demonstrated an effective scheme to tune the LC pitch [44–46]. In this work, an azobenzene chiral molecule [47] (CD-1) with large helical twisting power for highly sensitive photoresponsibility was adopted for its potential in achieving optically tunable LC lasers. Under UV/Vis irradiation, this molecule undergoes reversible *trans-cis* isomerization (Figure 3(a) and Fig. S9), leading to the modulation of the LC pitch and thus the shift of the corresponding photonic band edges. Efficient pitch modulation was verified by the transmission spectra of these photoresponsive CD-doped LCs, where obvious shift of the photonic band edge was observed under temporal UV (365 nm) illumination (Figure 3(b)). Consequently, the lasing wavelength of the LC microunit was effectively tuned to the bluer position (601 nm) (Figure 3(c)). Under the exposure to a visible light (532 nm), the lasing wavelength of the microunit was recovered (637 nm) due to the *trans*-isomerization of CD-1. In this way, the lasing wavelength of the microunit can be reversibly switched from 637 nm to 601 nm by altering the 365 nm and 532 nm light irradiations. The two lasing wavelengths remain nearly unchanged after six cycles (Figure 3(d)), demonstrating good reproducibility and tolerance with low device fatigue. It is worth noting that apart from the photoresponsive modulation, lasing wavelength could also move back to longer-wavelength position by itself after about 9 hours (Fig. S10) since the photoresponsive molecule CD-1 can undergo thermal relaxation to a more stable *trans* state [47], which, however, is too long for conceptual and practical information encryption applications. Moreover, the wavelength-switchable lasing behavior of LC microunits can be maintained in several days owing to the well capsulation of the MgF_2_ wafers (Fig. S11), which verifies the good durability of LC microlasers in ambient condition. This optically controlled modulation of the lasing wavelength of the microunit affords an efficient authentication method for confidential information encryption.

**Figure 4:**
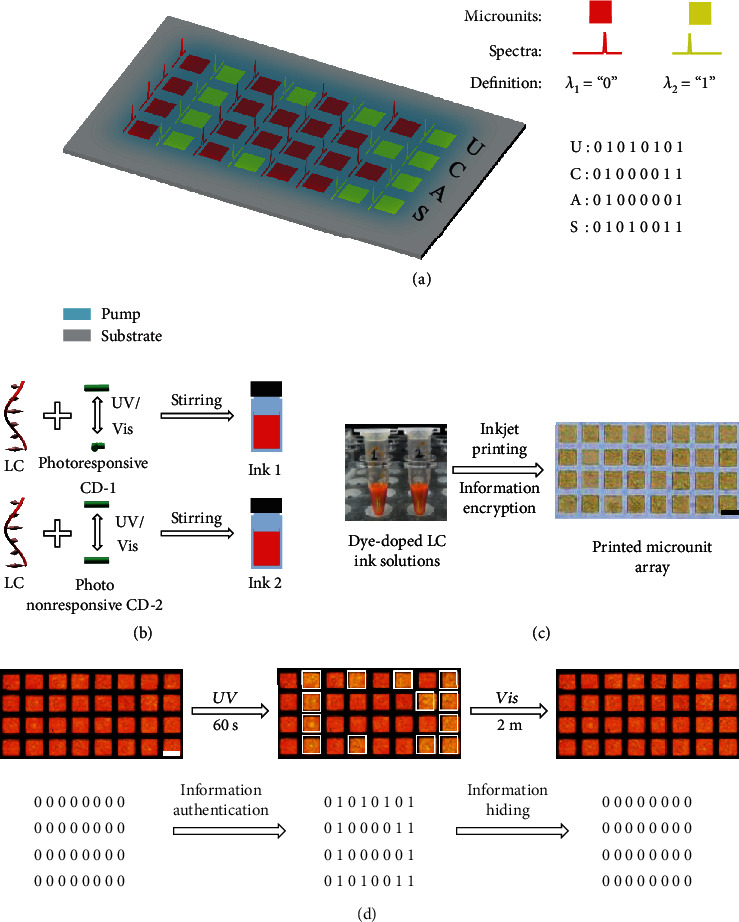
Confidential information encryption and authentication based on a phototunable LC microlaser array. (a) Concept of information encryption with patterned LC microlasers serving as cryptographic primitives. (b) Schematic illustration for the preparation process of chiral dopant-doped LC inks. Ink 1 and ink 2 are prepared by dissolving chiral dopants of photoresponsive CD-1 and photo-nonresponsive CD-2 into the LC matrix, respectively. (c) Information encryption process, which is carried out by encoding the raw information in a LC microlaser array via inkjet printing. Scale bar: 50 *μ*m. (d) Information authentication and hiding processes on a LC microlaser array. The LC microlasers, whose wavelengths represent the binary code of “1,” are depicted as the white squares in the middle panel. Scale bar: 50 *μ*m.

As a proof-of-concept encryption application, we exploited LC microlasers as cryptographic primitives for information encryption, with their wavelengths representing binary codes to encrypt meaningful information. Remote and relatively fast optical modulation, instead of 9-hour-period thermal relaxation modulation of lasing wavelength, was selected as the mechanism for the demonstration of minute-level confidential encryption and decryption. According to the ASCII binary codes, an arbitrary character can be translated into a standard 8-bit binary code sequence, e.g., “01010101” for the capital letter “U.” Thus, specific raw information composed of a number of English characters, for instance, “UCAS” (acronym of University of Chinese Academy of Sciences), can be effectively encrypted in a series of LC microlasers, with two of their lasing wavelengths representing “0” and “1,” respectively (Figure 4(a)). In this work, two kinds of inks, ink 1 and ink 2, were prepared by incorporating photoresponsive and photo-nonresponsive chiral dopants into LC matrixes, respectively (Figure 4(b)). Information encryption was carried out by encoding the raw information in an 8 × 4 LC microlaser array via inkjet printing (Figure 4(c)). The bright-field photograph of the microlaser array suggests that the information is efficiently encrypted, despite the fact that these patterned microlasers were prepared with different inks. Upon an integral excitation (Fig. S12), laser emissions from individual microunits were generated, which are not visually distinguishable (Figure 4(d)), thus ensuring high security of the protected information. The initial wavelengths (637 nm) of those LC microlasers at specific positions shifted to bluer ones (601 nm) after UV illumination (Fig. S13), revealing the secret information of “UCAS” and this information was rehidden in the microlaser array after the illumination of a visible light, which demonstrates a prototype of confidential information encryption and authentication. These results manifest the applicability of wavelength-tunable LC microlasers as cryptographic primitives for high-security information protection.

## 3. Discussion

In conclusion, we have demonstrated confidential information encryption and authentication with phototunable LC microlaser arrays. The LC microlaser arrays were effectively fabricated via a microtemplate-assisted inkjet printing method, where single-mode lasing emissions from individual microunits were obtained. The photoisomerization of CDs permits the variation of pitches of LC superstructures and thus the modulation of the wavelength of LC microlasers. On this basis, we achieved high-security information encryption and authentication on LC microlaser arrays using the wavelength of the LC microlasers as primary codes. We postulate these results would provide valuable enlightenment to the development of wavelength-tunable microlaser-based information security and anticounterfeiting applications.

## 4. Materials and Methods

### 4.1. Materials

The liquid crystal (LC) (I32-010E-425) was purchased from Shijiazhuang Slichem Display Material Co. Ltd. and used as received without further treatment. 4-(Dicyanomethylene)-2-methyl-6-(4-dimethylaminostyryl)-4H-pyran (98%) was purchased from Sigma-Aldrich Co. and purified by sublimation in nitrogen atmosphere. The chiral dopant (CD) of CD-1 was synthesized according to Ref. [46]. 2-Methyl-1,4-phenylene bis(4-((6-(acryloyloxy)hexyl)oxy)benzoate) (C6M) as a photopolymerizable monomer and 2,2-dimethoxy-2-phenylacetophenone (Irgacure 651) as a photoinitiator for UV curing to capture LC pitches were purchased from Beijing Innochem Science & Technology Co. Ltd. Araldite 506 epoxy resin was purchased from Sigma-Aldrich Co. The chiral dopant of CD-2 (S811) was purchased from Shijiazhuang Slichem Display Material Co. Ltd. and purified by sublimation in nitrogen atmosphere.

### 4.2. Fabrication of Liquid Crystal Microlaser Arrays

The polymeric microtemplates were prepared by etching polymeric films on substrates via electron beam lithography (EBL). In this work, MgF_2_ wafers were chosen as the substrates because of their low reflective index (1.38), which can reduce optical loss from the substrates and lower the threshold of laser emissions. The polymeric films were obtained by spin coating a poly(methyl methacrylate) (PMMA) solution (20 wt%, in chlorobenzene) on the pretreated (oxygen plasma, 5 min) substrates at 1200 rpm for 1 min and baked on a heating stage at 180°C for 5 min to eliminate the solvent. Then, electron beam exposures were carried out by an e-beam writer (ELPHY Quantum; Raith GmbH, Germany) at 30 kV with the beam-spot size of 3.0 nm, the beam current of 120 pA, and the patterning dose of 0.5 mC/cm^2^. Finally, the sample was developed in the mixed solution of methyl isobutyl ketone and isopropyl alcohol (1 : 3) for 30 s.

The LC-filled microunits were obtained by depositing inks into the polymeric microtemplates using a GIX™ Microplotter™ II from SonoPlot Inc. The cholesteric LC inks (ink 1 and ink 2) were prepared by dissolving 15 mg of CD-1 and 25 mg of CD-2 (S811) in the LC (I32-010E-425) matrix, respectively. The printing process included imbibing ink solutions with a hollow glass needle via capillary action and depositing the solutions into prefabricated microtemplates assisted by ultrasonic vibration. By adjusting the ultrasonic vibration strength, ink solution perfectly filled microunits were obtained.

The microunit laser arrays were fabricated by sequentially depositing luminescent inks into polymeric microtemplates on the substrate according to predesigned digital patterns. The red-emissive inks were prepared by dissolving 4.5 mg each of the dye in 300 mg of LC solution (1.5 wt%). The inks (ink 1 and ink 2) were printed into the microtemplates at required positions to produce 8 × 4 microlaser arrays. Before printing, the glass needle was cleaned by washing (dichloromethane) to avoid ink contamination.

### 4.3. Characterization

The morphology of the polymeric microtemplates was examined by a step profiler (Bruker Dektak XT) and a contourgraph (Bruker Contour GT-K1). The superstructure pitch characteristics were examined with scanning electron microscopy (Hitachi SU8010). The absorption and fluorescence spectra were measured by a UV-visible spectrometer (Perkin-Elmer Lambda 35) and a fluorescent spectrometer (Hitachi F-7000), respectively. Bright-field and fluorescence microscopy images were taken using an inverted fluorescence microscope (Nikon Ti-U) by exciting the samples with the halogen and mercury lamps, respectively. The optically pumped lasing measurements for individual microunits were carried out on a custom microphotoluminescence system. The excitation pulses (490 nm) were generated from an optical parametric amplifier (Light Conversion TOPAS) pumped by a regenerative amplifier (Spectra Physics, 800 nm, 150 fs, 1 kHz), which was in turn seeded by a mode-locked Ti : sapphire laser (Mai Tai, Spectra Physics, 800 nm, 150 fs, 80 MHz). The excitation laser was filtered with a 500 nm short-pass filter and then focused down to a 20 *μ*m diameter spot through an objective lens (Nikon CFLU Plan, ×20, N.A. = 0.5) as a nearly uniform pump source. The power at the input was altered by a neutral density filter. The emissions from the individual microunits were collected by the same objective with a back-scattering configuration and analyzed by the spectrometer after removing the excitation beam with a 500 nm long-pass filter.

## Supplementary Materials

Supplementary Materials Fig. S1: three-dimensional profiles of the microunits fabricated under different vibration strengths. Fig. S2: bright-field and photoluminescence microscopy images of the microunits with different shapes. Fig. S3: bright-field and photoluminescence microscopy images of the microunits with different sizes. Fig. S4: superstructure pitch characterization of the liquid crystals. Fig. S5: spectral data of the laser dye. Fig. S6: schematic demonstration of the experimental setup for lasing characterization. Fig. S7: photoluminescence spectra of the microunit under different pump fluences. Fig. S8: photoluminescence spectra of the dye in the microunit filled with epoxy resin. Fig. S9: molecular structure and absorption spectra of the chiral dopant under UV illumination. Fig. S10: the plot of the wavelength of the liquid crystal microlaser vs. the time under dark condition. Fig. S11: the plot of the wavelength modulation of the liquid crystal microlaser vs. the time. Fig. S12: schematic illustration of the experimental approach for the integral excitation of the microunit array. Fig. S13: photoluminescence spectra of the microunit array shown in Figure 4(d).
