# Supplementary material for: Wavelength-Tunable Single-Mode Microlasers Based on Photoresponsive Pitch Modulation of Liquid Crystals for Information Encryption
Source: Research (Wash D C). 2020 Dec 2;2020:6539431. doi: 10.34133/2020/6539431 (PMC7877376; doi:10.34133/2020/6539431)
Supplement: Supplementary Materials — Fig. S1: three-dimensional profiles of the microunits fabricated under different vibration strengths. Fig. S2: bright-field and photoluminescence microscopy images of the microunits with different shapes. Fig. S3: bright-field and photoluminescence microscopy images of the microunits with different sizes. Fig. S4: superstructure pitch characterization of the liquid crystals. Fig. S5: spectral data of the laser dye. Fig. S6: schematic demonstration of the experimental setup for lasing characterization. Fig. S7: photoluminescence spectra of the microunit under different pump fluences. Fig. S8: photoluminescence spectra of the dye in the microunit filled with epoxy resin. Fig. S9: molecular structure and absorption spectra of the chiral dopant under UV illumination. Fig. S10: the plot of the wavelength of the liquid crystal microlaser vs. the time under dark condition. Fig. S11: the plot of the wavelength modulation of the liquid crystal microlaser vs. the time. Fig. S12: schematic illustration of the experimental approach for the integral excitation of the microunit array. Fig. S13: photoluminescence spectra of the microunit array shown in Figure 4(d). [file 6539431.f1.pdf]

Supplementary Materials for

Wavelength-Tunable Single-Mode Microlasers Based on Photo-Responsive Pitch Modulation of Liquid Crystals for Information Encryption

Fa-Feng Xu, Zhong-Liang Gong, Yu-Wu Zhong, Jiannian Yao, and Yong Sheng Zhao*

This PDF file includes:

Fig. S1. Three-dimensional profiles of the microunits fabricated under different vibration strengths.

Fig. S2. Bright-field and photoluminescence microscopy images of the microunits with different shapes.

Fig. S3. Bright-field and photoluminescence microscopy images of the microunits with different sizes.

Fig. S4. Superstructure pitch characterization of the liquid crystals.

Fig. S5. Spectral data of the laser dye.

Fig. S6. Schematic demonstration of the experimental setup for lasing characterization.

Fig. S7. Photoluminescence spectra of the microunit under different pump fluences.

Fig. S8. Photoluminescence spectra of the dye in the microunit filled with epoxy resin.

Fig. S9. Molecular structure and absorption spectra of the chiral dopant under UV illumination.

Fig. S10. The plot of the wavelength of the liquid crystal microlaser *vs* the time under dark condition.

Fig. S11. The plot of the wavelength modulation of the liquid crystal microlaser *vs* the time.

Fig. S12. Schematic illustration of the experimental approach for the integral excitation of the

microunit array.

Fig. S13. Photoluminescence spectra of the microunit array shown in Fig. 4d.

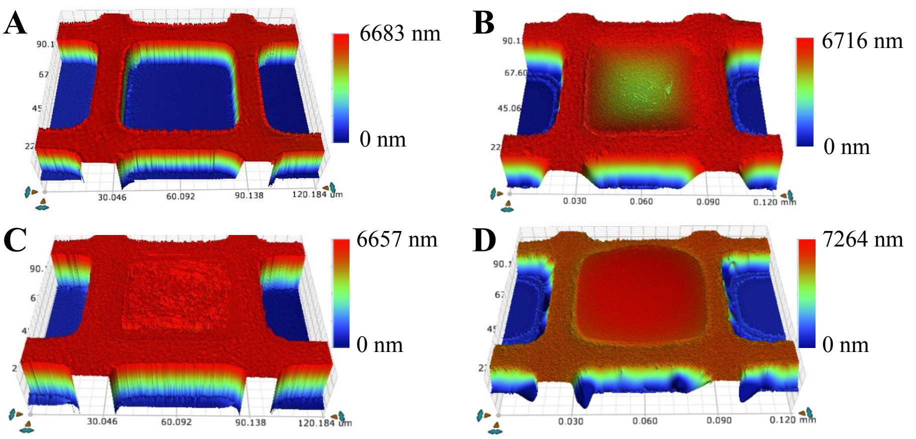

Fig. S1. Three-dimensional profiles of the microunits fabricated under different vibration strengths. (**A-D**) Three-dimensional profiles of the microunits fabricated under applied vibration voltages of 0 V (A), 0.6 V (B), 0.8 V (C) and 1.0 V (D), respectively.

The microunits shown in Figure S1 were fabricated by applying different vibration voltages in the printing process. The vibration voltage is proportional to the deposited amount of LC-contained ink solutions. By applying an appropriate vibration voltage of 0.8 V, an ink solution perfectly filled microunit was obtained, as depicted in Figure S1C. This result demonstrates that the volume of LC-contained ink solutions can be finely tuned by varying the vibration voltage, which ensures the successful construction of LC perfectly filled microunits.

A
B
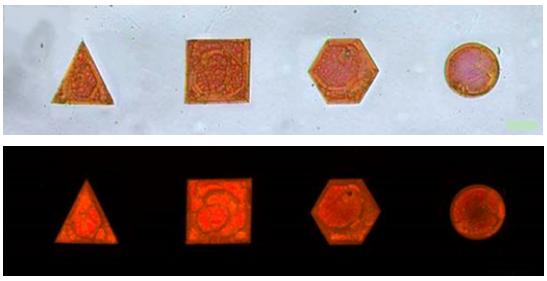

Fig. S2. Bright-field and photoluminescence (PL) microscopy images of the microunits with different shapes. (**A**, **B**) Bright-field (A) and PL (B) microscopy images of the microunits with different shapes, including triangle, square, hexagon, and circle. The photoluminescence image of these microunits was acquired under UV (330-380 nm) excitation. Scale bar: 50 µm. This result demonstrates that the microtemplate-assisted inkjet printing is a reliable method for the fabrication of microunits.

A

B

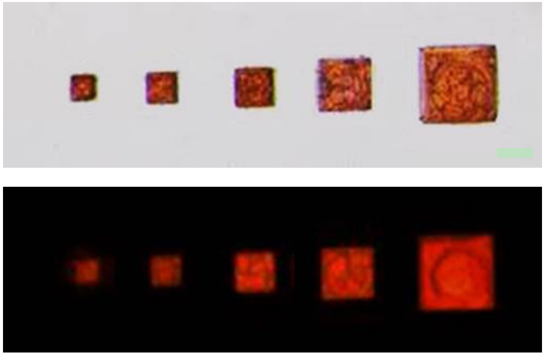

Fig. S3. Bright-field and PL microscopy images of the microunits with different sizes. (**A**, **B**) Bright-field (A) and PL (B) microscopy images of the microunits with different sizes. The PL image of the microunits was acquired under UV (330-380 nm) excitation. Scale bar: 50 μm. This result demonstrates that microunits with desired sizes can be effectively acquired through the microtemplate-assisted inkjet printing method.

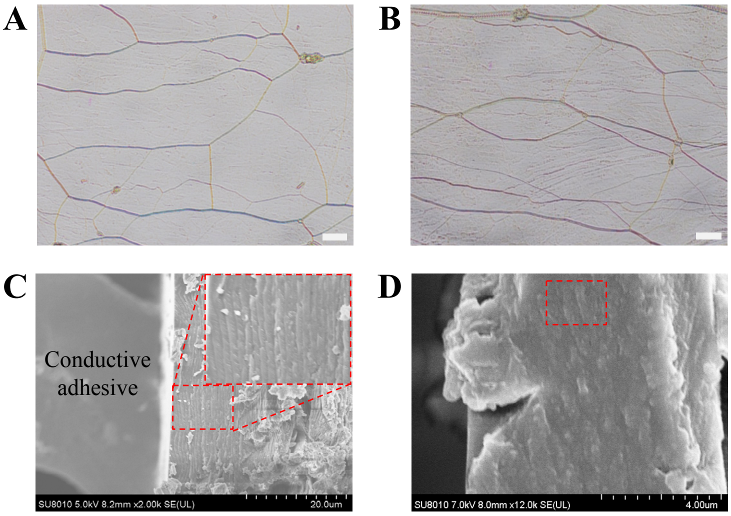

Fig. S4. Superstructure pitch characterization of the liquid crystals. (**A**, **B**) Bright-field microscopy images of two kinds of LC-contained ink solutions: ink 1 (A) and ink 2 (B). Scale bars: 100 μm. (**C**) SEM image of fractured surface of LC sample in a cell (wt%: LC-contained ink/C6M/Irgacure 651 = 33%/66%/1%) after UV curing (20 mW cm^{-2}, 365 nm) at 66 ℃ for 10 minutes. Inset: Corresponding enlarged image. (**D**) SEM image of fractured surface of LC sample in a microunit (wt%: LC-contained ink/C6M/Irgacure 651 = 33%/66%/1%) after UV curing (20 mW cm^{-2}, 365 nm) at 66 ℃ for 10 minutes.

As depicted in Fig. S4A and S4B, apart from the defect lines, LC molecules are believed to self-assembly assisted by chiral dopant molecules and form planar alignment in the clear areas where ordered superstructures dominate. This speculation has been proofed by the SEM images of LCs not only in a cell (Fig. S4C) but also in a microunit (Fig. S4D). In the cell, the inner surfaces of the top and bottom substrate are believed to play an important role in inducing the initial attachment and alignment of liquid crystal molecules, while in the microunit, polymeric microtemplates probably assist the ordered arrangement of LC molecules [30]. The formation of LC superstructures means LC molecules can provide distributed feedback cavities for optical oscillation for laser emissions [38].

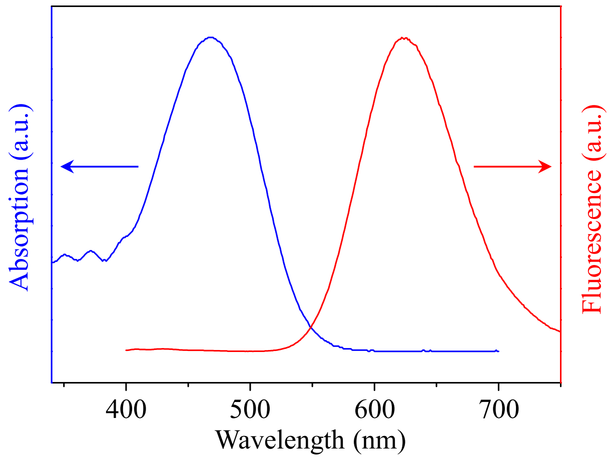

Fig. S5. Spectral data of the laser dye. Normalized absorption and emission spectra of the dye in ethanol solutions. The concerntration of the dye is 0.01 mM.

The emission spectra of the dye covered the red waveband range of 580-700 nm, offering an opporunity to achieve red-emissive lasers.

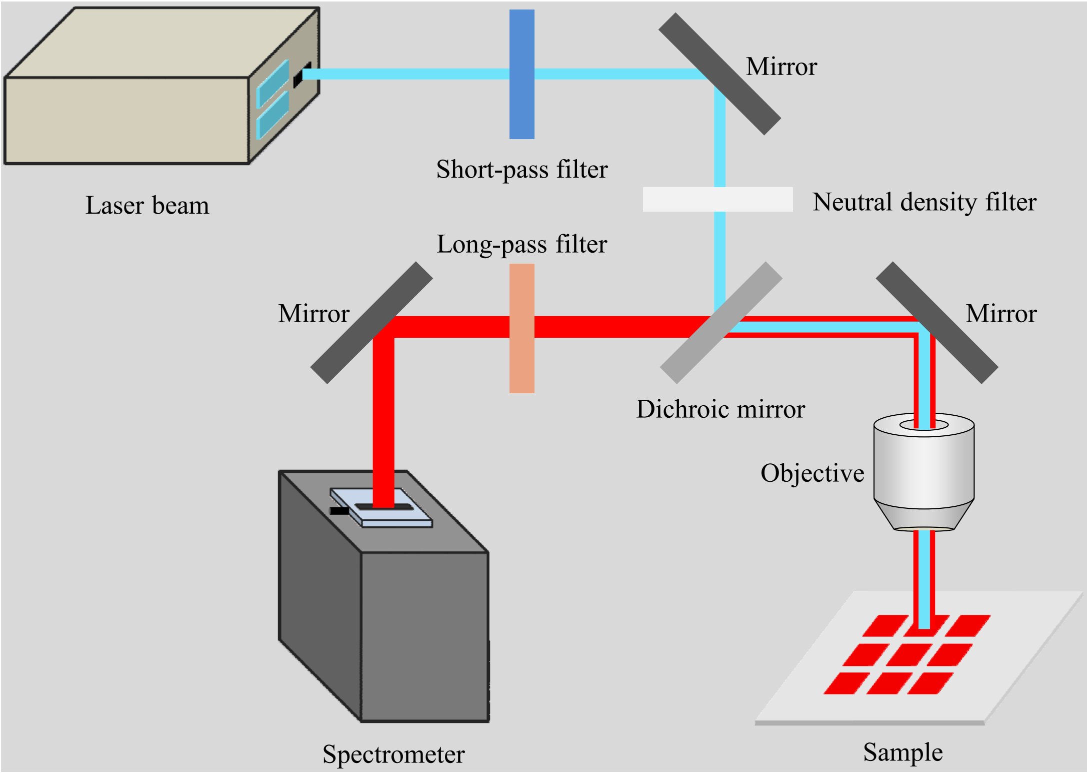

Fig. S6. Schematic demonstration of the experimental setup for lasing characterization.

The lasing performances of the fabricated microunits were examined on a custom micro-photoluminescence system. The 490-nm femtosecond laser (Spectra Physics) was employed as the excitation source, and the excitation energy was altered by a neutral density filter. The PL signal was collected with an objective lens (Nikon CFLU Plan, 20 ×, N.A. = 0.5). After passing through the corresponding filters (500-nm long-pass), the collected emissions were dispersed with a grating (1200 G mm^{-1}) and recorded by a monochrometer (Princeton Instrument Acton SP2300i) connected with a thermal-electrically cooled CCD (Princeton Instrument ProEM: 1600B).

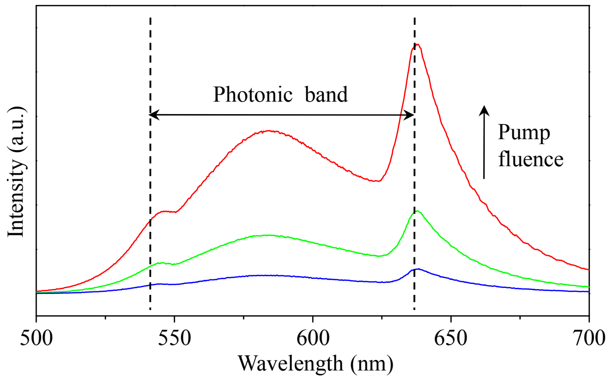

Fig. S7. Photoluminescence spectra of the microunit under different pump fluences.

The density of optical state (DOS) plays a key role in achieving laser emissions in dye-doped LC systems. According to the DOS distribution theory [38], DOS diverges at the edges of the photonic band due to the fact that the group velocity approches zero in proximity of the edges and the resulting long dwell time of the emitted photons strongly supports stimulated emission [38]. Consistent with this theory, we experimentally found that in the fabricated microunit, PL spectra of the laser dye were obviously modulated by the photonic band of the LCs, with much higher PL emissions at the long-wavelength bandedge (~637 nm) than that inside the photonic band and this spectral modulation was more evident as increasing the pump fluence. These results demonstrate the DOS distribution significantly modulates the PL spectra of the microunit and low-threshold lasing would be expected at the long-wavelength bandedge of LCs under sufficient pump fluence [44].

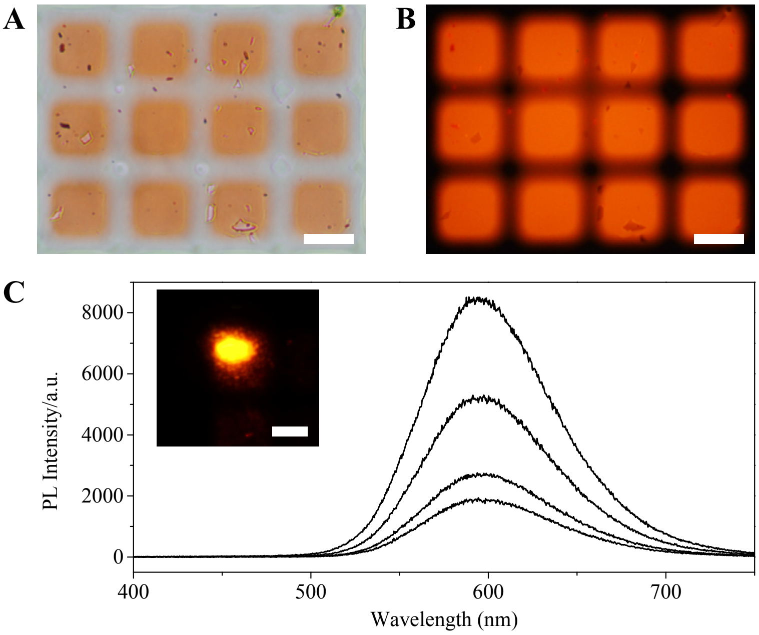

Fig. S8. Photoluminescence spectra of the dye in the microunit filled with epoxy resin. (**A**, **B**) Bright-field (A) and PL (B) mciroscopy images of the microunit array fabricated with dye-doped epoxy resin. Scale bars: 50 µm. (**C**) PL spectra of a microunit filled with dye-doped epoxy resin under the excitation of a fs laser. Inset: Corresponding PL image under the excitation. Scale bar: 20 µm.

As shown in Fig. S8, the broadband peaks of the microunit under increasing pump fluence originate from the spontaneous emission of the dye, which is in stark contrast with narrow-band lasing peaks of the LC-contained microunit and thus benefit the demonstration of the superiority of the lasing peaks [40].

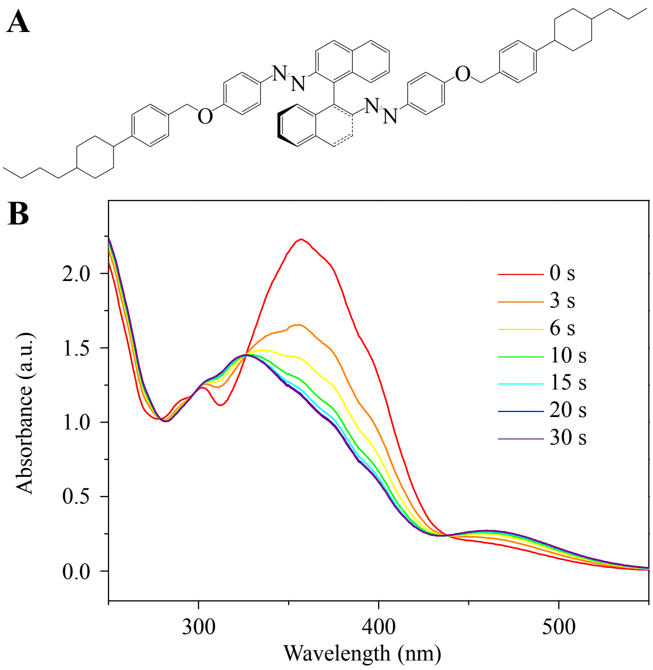

Fig. S9. Molecular structure and absorption spectra of the chiral dopant under UV illumination. (**A**) Molecular structure of the chiral dopant (CD-1). (**B**) UV-vis absorption spectra of CD-1 (40 μM) in CH_2Cl_2 under UV irradiation at 365 nm (20 mW cm^{-2}) with 0 s, 3 s, 6 s, 10 s, 15 s, 20 s and 30 s.

As depicted in Figure S9, the absorption spectra of CD-1 solutions in the ultraviolet waveband gradually decreased with increasing the time of UV illumination, which reveals that the chiral dopant undergoes the photochemical isomerization from (*trans*, *trans*)-CD-1 isomer to (*cis*, *cis*)-CD-1 isomer, thus providing an opportunity to modulate the pitch of LCs and corresponding wavelength of LC microlasers.

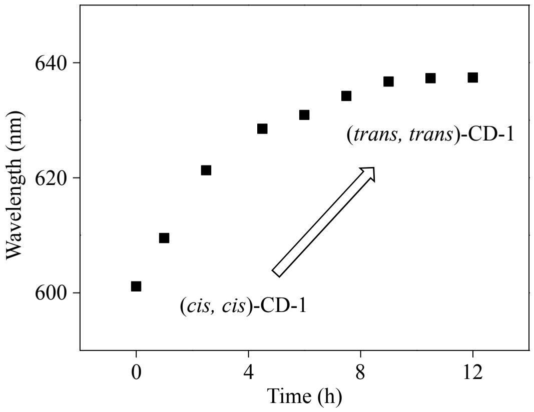

Fig. S10. The plot of the wavelength of the liquid crystal microlaser *vs* the time under dark condition.

In dark condition, the chiral dopant (CD-1) in the LC matrix underwent thermal relaxation to a more thermodynamically stable *trans* state by itself, which expanded the pitch of LC superstructures because CD-1 in *trans* state processes a relatively larger spatial configuration and thus shifted the position of the bandedge of LCs [45]. The modulation of the bandedge of LCs consequently led to the variation of the wavelength of the LC microlaser, which moved to redder positions until the configuration transformation of CD-1 was accomplished. This result demonstrates that the wavelength of LC microlasers can be also modulated through thermal relaxation of CD-1 with a 9-hour period besides optical stimulation.

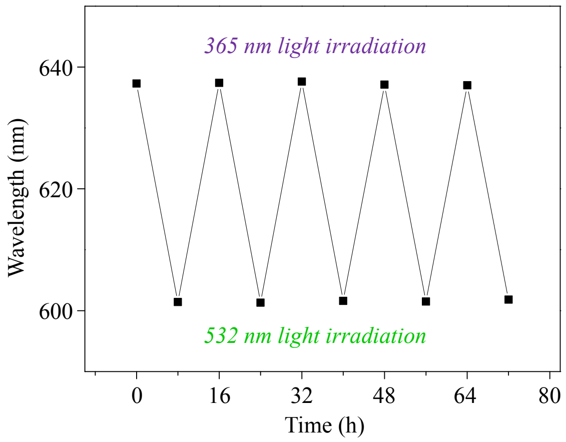

Fig. S11. The plot of the wavelength modulation of the liquid crystal microlaser *vs* the time. The emission spectra of the liquid crystal microlaser were collected after the alternative UV/Vis illuminations for 1 minute (365 nm, 20 mW cm^{-2}) and 2 minutes (532 nm, 20 mW cm^{-2}), respectively, between which there is an 8-hour interval.

The wavelength-switchable lasing behavior of the LC microlaser can be maintained after three days in ambient condition, which is attributed to the well capsulation of the MgF_2 wafers. This result demonstrates that the fabricated LC microlasers possess good durability.

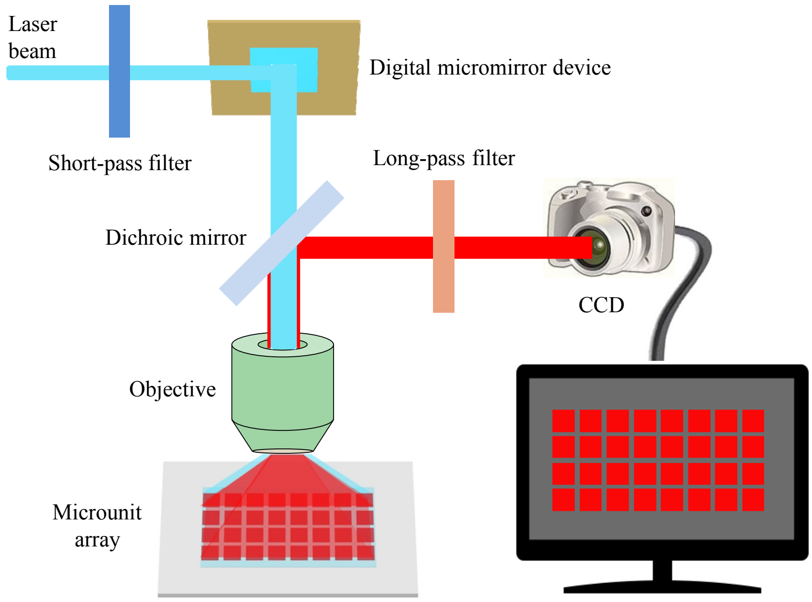

Fig. S12. Schematic illustration of the experimental approach for the integral excitation of the microunit array. The laser beam was expanded by a digital micromirror device to fully lighten up patterned microunits. The pump fluence was altered by a neutral density filter from 0 to 50 μJ cm^{-2}.

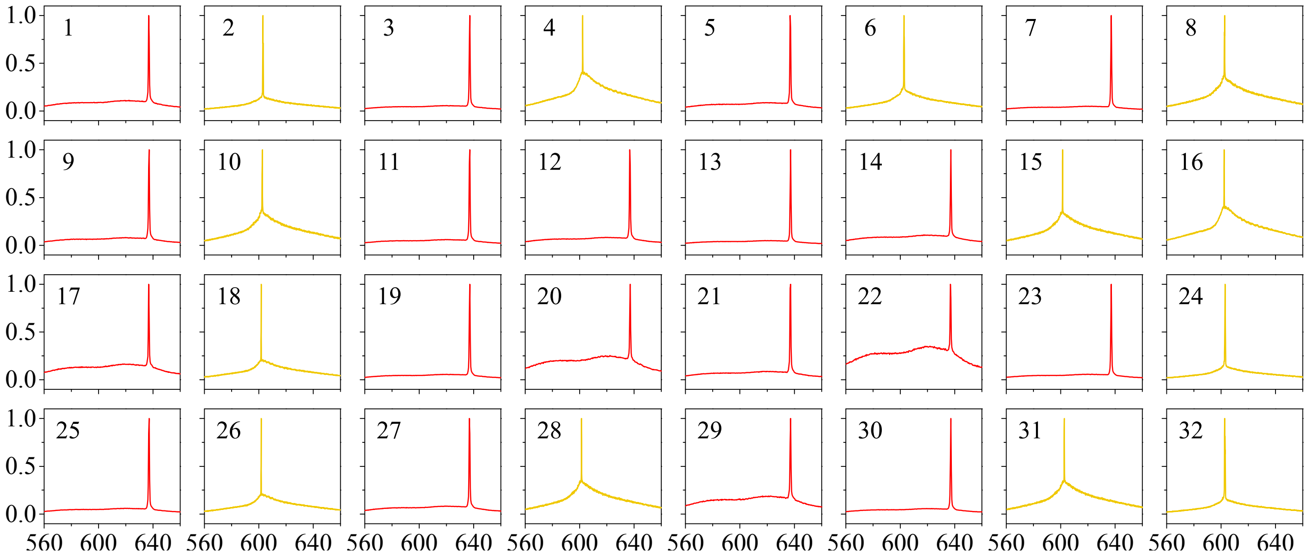

Fig. S13. Photoluminescence spectra of the microunit array shown in Fig. 4d. In each subplot, the horizontal axis measures wavelength (in nanometres) and the vertical axis represents the normalized photoluminescence intensity.
